# HTLV-1/2 prevalence in Brazilian blood donors: regional and demographic variation

**DOI:** 10.1186/1742-4690-8-S1-A83

**Published:** 2011-06-06

**Authors:** Anna B F Carneiro-Proietti, Ester C Sabino, Silvana Leão, Paula Loureiro, Moussa Sarr, Michael Busch, Fernando A Proietti, Edward L Murphy

**Affiliations:** 1 Hemominas Foundation, Brazil; 2 Pró-sangue Foundation, Brazil; 3 Hemope Foundation, Brazil; 4 Westat, USA; 5 University of California, San Francisco, California, USA; 6 Federal University of Minas Gerais, Minas Gerais, Brazil

## Background

HTLV-1/2 infection is endemic in Brazil and testing in blood donors has been mandatory since 1993. Several studies show that the infection is more prevalent in women and occurs in clusters of higher prevalence. However recent data from a representative national study of Brazilian blood donors is lacking.

## Methods

As part of a research network of blood centers, we studied all first-time blood donations in 2007 through 2009 from Fundação Pró-Sangue (FPS) in São Paulo State (Southeast), Hemominas, in Minas Gerais State (Southeast) and Hemope in Pernambuco State (Northeast) in Brazil. Serum samples were tested with two EIAs for HTLV-1/2. A validation study using the 2007 samples confirmed good correlation between this strategy and Western Blot; 90% of positive samples were HTLV-1. Prevalence and 95% confidence intervals were calculated, and adjusted odds ratios (aORs) were derived from logistic regression.

## Results

From 281,760 first-time donors tested for HTLV, 363 were positive (prevalence 129 per 100,000, 95% CI 116-142 per 100,000) on both EIA’s. Prevalence differed by region, ranging from 79 in Minas Gerais to 101 in São Paulo and 206 per 100,000 in Pernambuco, with little variation over the three years of the study. Prevalence increased with age, rising from 45 (age <20) to 221 per 100,000 (age 50+) in men and from 62 (age <20) to 282 per 100,000 (age 50+) among women. Prevalence was higher among those with black skin color (207 per 100,000) versus mixed (148 per 100,000) or white (76 per 100,000). In the logistic regression model, significant associations were found between higher HTLV-1/2 seropositivity and increasing age (aOR=5.00 for age 50+ vs. <20), female sex (aOR=1.84), black (aOR=2.81 vs. white) and Mixed skin colors (aOR=1.94 vs. White) and inversely with education (aOR=0.51 for college vs. less than high school education). There was no significant association with volunteer vs. replacement donor status.

## Conclusion

These results show Brazilian blood donor prevalence of HTLV-1/2 on the order of one per thousand, with regional differences probably due to ethnic origin of the underlying population. Demographic and socioeconomic differences are similar to those previously described, and are probably related to variations in sexual and maternal-child transmission. Blood center networks can provide useful surveillance data to monitor secular and geographic trends in HTLV-1 prevalence.

